# The Association between Somatotropin Therapy and the Risk of SARS-CoV-2 Infection in Children with Short Stature: A Population-Based Cross-Sectional Study

**DOI:** 10.3390/children9121844

**Published:** 2022-11-28

**Authors:** Gherta Brill, Iris Manor, Roberta Bril Paroz, Shai Ashkenazi, Shira Cohen, Avivit Golan-Cohen, Ilan Green, Ariel Israel, Shlomo Vinker, Abraham Weizman, Eugene Merzon

**Affiliations:** 1 Pediatric Endocrinology Unit, Leumit Health Services, Tel-Aviv 6473817, Israel; 2 ADHD Unit, Geha Mental Health Center, Clalit Health Services, Petah Tikva 49100, Israel; 3 Department of Psychiatry, Sackler School of Medicine, Tel Aviv University, Tel Aviv 6997801, Israel; 4 Sackler Faculty of Medicine, Tel Aviv University, Tel Aviv 6997801, Israel; 5 Adelson School of Medicine, Ariel University, Ariel 4077625, Israel; 6 Department of Family Medicine, Sackler Faculty of Medicine, Tel Aviv University, Tel Aviv 6997801, Israel; 7 Leumit Health Services, Medical Division, Tel Aviv 6473817, Israel

**Keywords:** growth hormone, growth hormone deficiency, idiopathic short stature, COVID-19, SARS-CoV-2, immune system, ADHD, short stature, somatotropin

## Abstract

COVID-19 is a worldwide pandemic caused by SARS-CoV-2, to which adults are usually more susceptible than children. Growth hormone (GH) levels differ between children and adults and decrease with age. There is bidirectional crosstalk between the GH/insulin-like growth factor-1 (IGF-1) pathway and the immune system that plays a significant role in SARS-CoV-2 infection. We evaluated the association between somatotropin treatment (GH replacement therapy) and the risk for SARS-CoV-2 positivity (a marker for COVID-19 infection) in children with growth hormone issues (GHI): growth hormone deficiency (GHD) and idiopathic short stature (ISS). A population-based cross-sectional study in Leumit Health Services (LHS) was performed using the electronic health record (EHR) database. The rates of SARS-CoV-2 positivity were evaluated among children with GHI, treated or untreated with somatotropin. Higher rates of SARS-CoV-2 positivity were found in GHI children, influenced by the same confounders reported in the pediatric population. A lower prevalence of SARS-CoV-2 PCR positivity was found among the somatotropin-treated children. A multivariate analysis documented that somatotropin treatment was associated with a reduced risk of SARS-CoV-2 positivity (Odds Ratio (OR) = 0.47, Confidence Interval (CI) 0.24–0.94, *p* = 0.032). Thus, somatotropin might be a protective factor against SARS-CoV-2 infections, possibly related to its immunomodulatory activity.

## 1. Introduction

The coronavirus disease 2019 (COVID-19) pandemic has been caused by the severe acute respiratory syndrome coronavirus 2 (SARS-CoV-2). It was first reported in Wuhan, China, and within a few months spread worldwide, causing, as of August 2022, >590 million cases and ~6.44 million deaths [1]. World Health Organization (WHO) data have demonstrated that adults and the elderly are more susceptible to severe disease and complications than children and adolescents [2]. The pediatric population commonly exhibits an asymptomatic or mild course of infection, often with atypical clinical manifestations [3,4]. The occurrence of severe and life-threatening illnesses in this population is rather scarce [5]. Moreover, it is estimated that in the younger than 20 years age group, susceptibility to the disease is about half of that in adults [6].

It is therefore important to elucidate the factors that might be related to the higher vulnerability of the older population to the infection. A couple of factors have been proposed [7], yet much uncertainty still remains.

Children generally have higher growth hormone (GH, also called somatotropin) levels than adults. Normally, daily GH secretion peaks at puberty and decreases gradually, with a progressive decline of approximately 15% during each decade [8]. Thus, we examined the role of GH treatment in SARS-CoV-2 infection susceptibility.

There are two main endocrine determinants of short stature (growth hormone issues (GHI)) linked to the GH axis: either inadequate secretion of endogenous GH, which is known as GH deficiency (GHD), or idiopathic short stature (ISS). ISS refers to very short children who do not always have an identifiable disorder of the GH/insulin-like growth factor-1 (IGF-1) axis and with no evidence of an endocrine, metabolic, or other abnormality that explains the short stature, but who show a response to growth hormone treatment [9].

A bidirectional crosstalk exists between the GH/IGF-1 pathway and the immune system [10,11,12,13,14,15]. Somatotropin administration to non-GH deficient children with short stature has been associated with increased levels of serum pro-inflammatory cytokines. These cytokines include interferon-gamma, interleukin (IL)-1-beta, IL-2, IL-12, and tumor necrosis factor (TNF)-alpha [13]. This finding indicates a possible effect of GH on immune function [11]. Other studies have documented that the cytokines TNF, IL-1, and IL-6 affects the GH/IGF-1 axis at several levels, with relevant consequences for human diseases [12,13]. GH has been shown to be a significant component, along with prolactin, for developing mature lymphocytes and maintaining immune competence [14]. Somatotropin therapy has also been reported to attenuate infectious processes in human immunodeficiency virus (HIV)-infected patients [15].

We hypothesized that children with GHI might exhibit higher susceptibility to SARS-CoV-2 positivity and that somatotropin treatment may have a protective role against this susceptibility. In the present study, we investigated the association between treatment with somatotropin in children with GHI and susceptibility to SARS-CoV-2 infection.

## 2. Materials and Methods

We conducted a retrospective population-based cross-sectional study. Data from the Leumit Health Services (LHS) database was utilized. LHS is a large nationwide health maintenance organization in Israel that provides services to approximately 725,000 members. The study period was from 1 February 2020 to 31 December 2020, before the start of the vaccination campaign against SARS-CoV-2 infection in Israel.

The LHS electronic health records (EHR) database is regularly updated concerning members’ demographics, visits, medical diagnoses, laboratory results, treatments, and hospitalizations. The somatic diagnoses are established during each medical visit according to the International Classification of Diseases (ICD-9) and the psychiatric diagnoses according to the ICD-10. All LHS members have the same health insurance coverage and access to healthcare services.

Data were retrieved with IBM Cognos 10.1.1 BI Report Studio software (IBM, Armonk, NY, USA). Query results were downloaded to Microsoft Excel (Version 14; Microsoft Corp., Seattle, WA, USA) spreadsheets for analysis. The statutory Research Committee of LHS and the Shamir Medical Center Institutional Review Board on Human Research approved the study protocol.

### 2.1. Study Population

All children (≤18 years) with GHI that were eligible for somatotropin therapy, who had been tested at least once for SARS-CoV-2 positivity during the study period, were included in the study. The criteria for GH therapy, as recommended by the FDA, include the following: (a) children with short stature and GHD who underwent two GH stimulation tests out of three available tests (clonidine, arginine, or glucagon) with peak GH levels below 7.5 ng/mL on both tests; (b) children with ISS without GHD who were shorter that the 1.2 percentile according to the CDC growth chart for age and sex and whose adult predicted height was below the normal range (150 cm in girls and 160 cm in boys) and who had no growth hormone deficiency and no endocrine or other disease that explains the short stature. The exclusion criteria were as follows: (1) the presence of chronic medical diseases that affect growth; (2) age > 18 years; (3) other conditions that are treated with somatotropin, like chronic renal failure, small for gestational age, as well as Turner, Noonan, and Prader–Willi syndrome.

The study population was divided into two groups: the treated group, defined as children who purchased at least one prescription of somatotropin during the year before the study period [16]; and the comparison group, which included the children who were eligible for GH treatment but were not treated with somatotropin, due to their parents’ decision.

### 2.2. Study Methods

Data regarding demographics, laboratory results, and diagnoses according to the ICD-9 for somatic diagnoses and the ICD-10 codes for psychiatric diagnoses were derived from the LHS electronic medical records (EMR) system. The children’s diagnoses were entered or updated during each physician visit. LHS records also use an ongoing diagnosis affirmation process: clinicians are encouraged to report to the medical division experts about patients they believe do not meet the criteria for a particular diagnosis. The diagnosis is checked and, if needed, removed.

Socioeconomic status (SES) was defined according to the Israeli Central Bureau of Statistics’ classification, which includes 20 subgroups. Classifications 1 to 7 were considered low SES, 8 to 13 were considered medium SES, and 14 to 20 were considered high SES.

Several diagnoses were reported as being associated with the risk of COVID-19 infection in children and were therefore defined as potential confounders. These included attention deficit hyperactivity disorder (ADHD) [17], diabetes mellitus [18], bronchial asthma [19], and vitamin D3 levels [20]. As influenza vaccination may constitute a confounder [21], it was controlled for in the current study. In Israel, influenza vaccination is offered free of charge to all residents older than six months, according to the National Vaccination Policy of 2012. Vaccinated children for the winter season of 2019–2020 were defined as influenza vaccinated.

We utilized the Israeli Ministry of Health criteria for the diagnosis of ADHD, following the international evaluation requirements (ICD-10). The diagnosing physician must be a senior physician specializing in the field of ADHD. The diagnosis is established according to the “American Psychiatric Association’s Diagnostic and Statistical Manual” (DSM-4 or -5, depending on the year of the diagnosis).

### 2.3. SARS-CoV-2 Testing

SARS-CoV-2 testing of samples derived from nasopharyngeal swabs was performed by experienced personnel in a centralized laboratory according to international guidelines. Testing was performed by a real-time polymerase chain reaction (RT-PCR) assay with internal positive and negative controls according to the guidelines of the World Health Organization. The Allplex™ 2019-nCoV Assay (Seegene Inc., Seoul, Republic of Korea) was used until 10 March 2020, and since then the COBAS COVID-196800/8800 (Roche Pharmaceuticals, Basel, Switzerland) was used.

### 2.4. Statistical Analysis

Statistical analysis was conducted using STATA 12 software (StataCorp LP, College Station, TX, USA). All tests were two-sided with significance set at 0.05. Student’s *t*-test and Fischer’s exact χ^2^ test were used as appropriate for continuous and categorical variables, respectively, based on the normal distribution and variable characteristics. Categorical data were expressed as rates. Continuous variables with normal distribution were defined as mean or percentile and 95% confidence interval (95% CI) or ±standard deviation (SD).

Stratified analyses included an initial univariate evaluation of risk estimates with subsequent multivariable regression models to examine crude and odds ratios (ORs) and CI for associations between somatotropin treatment and a positive PCR test for SARS-CoV-2 while controlling for the aforementioned potential confounders: age, gender, SES, previous influenza vaccination, low plasma vitamin D3 level (<20 ng/mL (50 nmol/L)) [22], and the presence of ADHD, diabetes mellitus, and asthma.

## 3. Results

The study population included 2382 children with GHI eligibility for GH therapy who were examined for SARS-CoV-2 infection. Of the study population, 421 (17.67%) tested positive for SARS-CoV-2 infection. Table 1 shows the sociodemographic and laboratory data of the SARS-CoV-2-positive vs. -negative children. The SARS-CoV-2-positive group was slightly older than the negative group (13.84 ± 3.91 vs. 13.02 ± 4.34 years, *p* = 0.038), with a higher rate of boys (69.83% vs. 59.31%, *p* = 0.001), and a higher percentage of low SES (71.50% vs. 49.31%, *p* = 0.001). They also had a higher body mass index (BMI) percentile (46.45 ± 34.35 vs. 42.38 ± 32.69, *p* = 0.0225) and lower plasma vitamin D3 levels (20.11 ± 7.86 vs. 23.40 ± 9.31, *p* = 0.001). The other parameters did not differ significantly between the groups.

Table 2 shows the clinical data, including medical and psychiatric comorbidities, in the study population. The SARS-CoV-2-positive group had higher rates of ADHD (28.27% vs. 20.91%, *p* = 0.001), vitamin D3 deficiency (27.32% vs. 19.12%, *p* = 0.001), and obesity (21.85% vs. 17.75%, *p* = 0.0488). In the SARS-CoV-2-negative group, there were higher rates of asthma (19.79% vs. 15.44%, *p* = 0.039), previous influenza vaccinations (15.96% vs. 9.26%, *p* = 0.0003), and somatotropin treatment (8.62% vs. 5.23%, *p* = 0.020).

The sociodemographic and clinical characteristics of somatotropin-treated vs. untreated children are presented in Table 3. There were higher rates of males in the somatotropin-treated group compared to the untreated group (69.1% vs. 60.5%, *p* = 0.018). Treated children had higher 25-OH vitamin D3 levels (25.1 ± 11.3 vs. 22.68.9, *p* = 0.006) and, as expected, had higher IGF-1 levels (273.3 ± 146.1 vs. 166.99 ± 108.4, *p* = 0.001). However, there were no significant differences in BMI, height and weight levels, age, SES, or comorbidities associated with SARS-CoV-2 positivity.

Table 4 shows the multivariate logistic regression analysis of the variables associated with the risk of SARS-CoV-2 positivity. Somatotropin treatment was significantly associated with a lower likelihood of SARS-CoV-2 positivity (adjusted OR = 0.47 (0.24–0.94), *p* = 0.032). The likelihood of infection with SARS-CoV-2 was also positively associated with male gender (adjusted OR = 1.83 (1.31–2.56), *p* = 0.006), low SES (adjusted OR = 2.03 (1.46–2.81), *p* = 0.001), ADHD diagnosis (adjusted OR = 1.60 (1.14–2.25), *p* = 0.032), obesity (adjusted OR = 1.58 (1.09–2.29), *p* = 0.022), and low plasma vitamin D3 levels (adjusted OR = 1.68 (1.23–2.31), *p* = 0.015).

## 4. Discussion

This study demonstrated that children with GHI who were treated with somatotropin exhibited a lower likelihood of SARS-CoV-2 infection compared to those who were untreated. This negative association remained significant after multivariate logistic regression analysis adjusted for several known confounders. This negative association between somatotropin treatment and SARS-CoV-2 positivity remained significant and independent after adjustment for several known factors, which may be associated both with the exposure (somatotropin treatment due to GHI) and the outcome (SARS-CoV-2 infection), such as age, gender, low SES, diagnosis of diabetes mellitus, diagnosis of asthma, diagnosis of ADHD, obesity, and low plasma vitamin D3 level. It has been confirmed that adults, especially the elderly, are generally more susceptible to infection by SARS-CoV-2, with severe presentation, complications, and mortality from COVID-19 infection, than children and adolescents [2,4,5]. As serum GH levels peak at puberty and steadily decrease thereafter, our finding of the plausible protective activity of somatotropin against COVID-19 infection may partially explain the susceptibility of adults and the elderly to this infection. The possible protective effect of somatotropin treatment was found to be independent of other factors. Our study is in line with previous publications demonstrating that male gender [23], high BMI [24], ADHD [17,25], and low 25-OH vitamin D levels [20] are risk factors for COVID-19 infection, while asthma [19] and influenza vaccination [21] are protective factors.

Notably, somatotropin treatment was found to be protective in all GHI children, including ISS and GHD children. Although the specific mechanisms involved in the protective effect are unclear, it may be related to the immunomodulatory effects of somatotropin. To our knowledge, this is the first study that has examined the role of somatostatin in the current global COVID-19 pandemic. It should be noted, however, that our results are in concert with a previous study demonstrating the possible protective immunomodulatory effect of somatotropin administration [26]. Further studies are needed to elucidate the precise mechanisms by which GH attenuates the susceptibility to COVID-19 infection and whether this effect is dependent upon GHD.

### Limitations

The present study has several limitations. First, it is an observational retrospective study that analyzed an electronic database. However, an advantage is the extensive national database that has been previously validated and the use of a comparative group, enabling the evaluation of potential confounders, and allowing the association to be determined by multivariate analysis. Second, we examined the association, rather than the causality, of such linkage. Moreover, unidentified confounders may play a role in reducing the rates of COVID-19 infection among somatotropin-treated children. Third, it is possible that somatotropin-treated children with short stature adhere more to social distancing and hygiene measures than the untreated children.

We also did not explore the symptomatology of the SARS-CoV-2 infection, its complications, and the need for hospitalization. These were beyond the scope of the current study. Indeed, it is suggested that the study findings should be repeated and confirmed in other geographic regions, maybe considering additional potential confounders.

## 5. Conclusions

Our findings indicate a negative association between somatotropin treatment of children with GHI and the likelihood of infection with SARS-CoV-2 during the COVID-19 pandemic. Notably, similar to observations in the general population [26], we also confirmed the association between higher risk for SARS-CoV-2-positive result and several cofounders, including male gender, low SES, obesity, ADHD, low plasma vitamin D3 levels, and lack of previous influenza vaccination, independently of the possible protective effect of the somatotropin treatment.

## Figures and Tables

**Table 1:** Demographic and laboratory characteristics of SARS-CoV-2 positive vs. SARS-CoV-2 negative subjects.

Variables		Total	SARS-CoV-2 Positive	SARS-CoV-2 Negative	*p*-Value
		2382 (100%)	421 (17.67%)	1961 (82.33%)	
Age years, mean ± SD		13.14 ± 4.29	13.84 ± 3.91	13.02 ± 4.34	0.038
Gender	Male	1457 (61.17%)	294 (69.83%)	1163 (59.31%)	0.001
	Female	925 (38.83%)	127 (30.17%)	798 (40.69%)	
SES	Low	1268 (53.23%)	301 (71.50%)	967 (49.31%)	0.001
	Middle	821 (34.47%)	108 (25.65%)	713 (36.36)	
	High	293 (12.3%)	12 (2.85%)	281 (14.33%)	
Height (percentile), Mean ± SD		12.31 ± 16.45	11.09 ± 16.41	12.57 ± 16.45	0.1005
Weight (percentile), Mean ± SD		23.95 ± 28.85	24.87 ± 29.37	23.76 ± 28.74	0.512
BMI (percentile), Mean ± SD		43.21 ± 33.04	46.45 ± 34.35	42.38 ± 32.69	0.0225
Glucose (mg/dL), Mean ± SD		86.84 ± 10.95	87.26 ± 10.61	86.74 ± 11.02	0.413
25-OH Vitamin D3 (ng/mL), Mean ± SD		22.81 ± 9.15	20.11 ± 7.86	23.40 ± 9.31	0.001
TSH (mIU/L), Mean ± SD		2.39 ± 1.33	2.41 ± 1.31	2.38 ± 1.34	0.6978
IGF-1 (ng/mL), Mean ± SD		175.52 ± 115.54	176.26 ± 105.61	175.35 ± 117.59	0.8844

SD—standard deviation; SES—socioeconomic status; BMI—body mass index; TSH—thyroid stimulating hormone; IGF-1—insulin-like growth factor 1.

**Table 2:** Clinical data, including medical and psychiatric comorbidities, in the study population.

Variable *N* (%)	Total	SARS-CoV-2 Positive	SARS-CoV-2 Negative	*p*-Value
	2382 (100%)	421 (17.67%)	1961 (82.33%)	
Diabetes mellitus	20 (0.84%)	6 (1.43%)	14 (0.71%)	0.056
Bronchial asthma	453 (19.02%)	65 (15.44%)	388 (19.79%)	0.039
Influenza vaccination 2019–2020	352 (14.78%)	39 (9.26%)	313 (15.96%)	0.0003
ADHD	529 (22.21%)	119 (28.27%)	410 (20.91%)	0.001
Low 25-OH vitamin D3 *	490 (20.57%)	115 (27.32 %)	375 (19.12%)	0.001
Missing data	1125 (47.23%)	196 (46.56%)	929 (47.37%)	
Obesity **	440 (18.47%)	92 (21.85%)	348 (17.75%)	0.0488
Somatotropin treatment	191 (8.02%)	22 (5.23%)	169 (8.62%)	0.0200

ADHD—attention deficit hyperactivity disorder; * low 25-OH vitamin D3 > 20 ng/mL; ** obesity—BMI percentile > 85%.

**Table 3:** Demographic and clinical characteristics of somatotropin-treated vs. untreated children.

Variables		Somatotropin Treated	Somatotropin Untreated	*p*-Value
		191 (8.02%)	2191 (91.98%)	
Mean age (years ± SD)		12.89 ± 3.67	13.06 ± 4.37	0.269
Gender	Male	132 (69.11%)	1325 (60.47%)	0.018
	Female	59 (30.89%)	866 (39.53%)	
SES	Low	104 (54.45%)	1164 (53.13%)	0.059
	Middle	60 (31.41%)	761 (34.73%)	
	High	27 (14.14%)	266 (12.14%)	
Height (percentile), Mean ± SD		13.03 ± 17.29	11.86 ± 15.46	0.326
Weight (percentile), Mean ± SD		20.59 ± 25.52	24.26 ± 29.16	0.856
BMI (percentile), Mean ± SD		42.86 ± 32.57	43.93 ± 33.05	0.482
25-OH vitamin D3 (ng/mL), Mean ± SD		25.14 ± 11.31	22.59 ± 8.89	0.006
IGF-1 (ng/mL), Mean ± SD		273.30 ± 146.15	166.99 ± 108.41	0.001
Bronchial asthma		42 (21.99%)	411 (18.76%)	0.275
Influenza vaccination 2019–2020		37 (19.37%)	315 (14.38%)	0.062
ADHD		47 (24.61%)	482 (22%)	0.405
Low 25-OH vitamin D3 *		77 (40.31%)	911 (41.58 %)	0.060
Missing data		84 (43.98%)	1048 (47.83%)	
Obesity **		28 (14.66%)	412 (18.80%)	0.157

ADHD—attention deficit hyperactivity disorder; * low 25-OH vitamin D3 > 20 ng/mL; ** obesity—BMI percentile > 85%; SD—standard deviation.

**Table 4:** Crude and adjusted OR (95%) of being positive for SARS-CoV-2.

		Crude OR			Adjusted OR		
Variables		OR	95% CI	*p*-Value	OR	95% CI	*p*-Value
Age categories	<6 (reference)	1					
	6–10 years	1.74	0.80–3.79	0.159	2.51	0.55–11.32	0.233
	10–15 years	2.73	1.30–5.73	0.008	2.44	0.56–10.57	0.232
	15+	3.92	1.88–8.17	0.001	3.96	0.93–16.92	0.063
Male gender		1.58	1.26–1.99	0.001	1.83	1.32–2.56	0.006
Low SES		2.57	2.04–3.24	0.001	2.03	1.46–2.811	0.001
Diabetes mellitus		2.01	0.96–5.26	0.055	2.17	0.60–7.82	0.232
Bronchial asthma		0.69	0.51–0.92	0.04	0.72	0.48–1.08	0.112
Influenza vaccination 2019–2020		0.54	0.38-–0.76	0.001	0.40	0.23–0.71	0.002
ADHD		1.49	1.17–1.89	0.001	1.60	1.14–2.25	0.032
Low 25-OH vitamin D3 *		2.00	1.30–3.07	0.002	1.68	1.23–2.31	0.015
Obesity **		1.29	1.001–1.68	0.049	1.58	1.09–2.29	0.022
Somatotropin treatment		0.58	0.37–0.92	0.021	0.47	0.24–0.94	0.032

OR—odds ratio; CI—confidence interval; SES—socioeconomic status; ADHD—attention deficit hyperactivity disorder; * low 25-OH vitamin D3 > 20 ng/mL; ** obesity—BMI percentile > 85%.

## Data Availability

Not applicable.
